# Carlos Américo Paiva Gonçalves Filho

**DOI:** 10.5935/0004-2749.20210089

**Published:** 2021

**Authors:** Harley E. A. Bicas

**Affiliations:** 1 Coordinator of the Directive and Administrative College of the Brazilian Council of Ophthalmology

Only few hours had passed since we welcomed the New Year - which was expected to offer a more normal way of life where social distancing was no longer necessary - when it brought an irrecoverable separation. Sadly I wish to express our appreciation and respect to one of the most expressive lifetime members of the Directive and Administrative College of the Brazilian Council of Ophthalmology (C.B.O.), Carlos Américo Paiva Gonçalves Filho. Among the past C.B.O.’s presidents alive, he had the honor of having the most antique presidential term (1975-1977) and was the only one to have served in office for a second time (1989-1991). He was also the president of the Brazilian Society of Ophthalmology (S.B.O., 1973-1974), and the 72^nd^ chairman of the National Academy of Medicine. Such eminent posts may suffice to give an idea of his brilliant academic career, since they implicitly make presumed meritorious recognition of excellence in a large number of other previously exerted duties.

However, greatness may be manifested by actions that may go unnoticed and seem simple in spite of having considerable semiotic meanings.

I am aware of it in Paiva Gonçalves. But, first of all, it is convenient to advance that he was a paradigmatic *carioca* (a native from Rio de Janeiro), always proud of showing his typical (and expected from *cariocas*) tanned skin, diligently acquired at the Rio`s beaches. *Cariocas* and *paulistanos* (natives from Sao Paulo city) besides of having quite different lifestyles were also developed to keep a legendary rivalry about their cities.

In 1974 Paiva Gonçalves completed his term as the president of the S.B.O. (which in spite of the title “Brazilian Society” had the meaning of being a pure “carioca’s society”), and in 1975, he became the president of the C.B.O. (which had its headquarters in São Paulo and, therefore, could be taken as “São Paulo”); thereafter, he used the Revista Brasileira de Oftalmologia (the official magazine of the S.B.O., the “carioca’s society”, as it had been called by *paulistanos*) to publicize the activities of the C.B.O. But to the Arquivos Brasileiros de Oftalmologia (the “São Paulo’s” ophthalmological magazine), in 1977, he bestowed a very decisive and definitive gift by officializing its financial support by the C.B.O. (since its foundation, in 1938, this magazine survival had been exclusively granted by the Belfort Mattos family, a typical “paulistanos’ family”). No provincialism, none alleged rivalry between C.B.O. and S.B.O., Paiva Gonçalves behaved not as a governor, but as a statesman to proclaim, without ostentation (as it is now customary to say): *I am, we are, all of us, “Brazilian Ophthalmology.”*

At the XIX Brazilian Congress of Ophthalmology (1977), of which he was the president, there were few Brazilian societies of ophthalmological “specialties,” namely the Brazilian Center of Strabismus (the first founded, in 1967 and, then, the most “powerful”), the Brazilian Society of Lenses of Contact (SOBLEC, 1971) plus the newborns Brazilian Society of Ocular Plastic Surgery (1975), and the Brazilian Society of Retina and Vitreous (1976). Such institutions used to promote private meetings during the larger event and charge appro priate taxes from the audience, taking advantages of the organization and settlements of the main Congress. With a rare, anticipatory discernment, Paiva Gonçalves was the first to impose the prohibition of such practices. The societies of ophthalmological “specialties” were displeased; however, this decision was and continues to be convenient.

Certain life circumstances did not allow me the oppor tunity to get to know him better. In fact, it is surprising that a man who has received so many meritorious homages and was still deserved of several others was so simple and decided to a modest asceticism, refusing to accept any new honors. I suppose he might have considered them superfluous, since the greatest one had already been received when he was a very young man, precisely from his so much loved (and famous) father. So that, I dare to quote the first, honorific, distinctive and premonitory pride dedicated from his father, who as early as 1958, advanced to him concluding that (why) “…just proud I inscribe this book”^(1)^; and, later on, by the admirable and renowned “Oftalmologia” of 1979, reiterating the loved homage, then with the book’s additive dedication that “This time it is to a winner that I inscribe to”^(2)^.
